# Highly Sensitive Determination of Antibiotic Residues in Aquatic Products by High-Performance Liquid Chromatography–Tandem Mass Spectrometry

**DOI:** 10.3390/antibiotics11101427

**Published:** 2022-10-17

**Authors:** Hongli Ye, Siman Li, Yinfeng Xi, Yongfu Shi, Xiaorui Shang, Dongmei Huang

**Affiliations:** 1 Laboratory of Aquatic Product Quality, Safety and Processing, East China Sea Fisheries Research Institute, Chinese Academy of Fishery Sciences, Shanghai 200090, China; 2 Key Laboratory of Control of Safety and Quality for Aquatic Product, Ministry of Agriculture and Rural Affairs, Beijing 100141, China; 3 College of Food Sciences and Technology, Shanghai Ocean University, Shanghai 201306, China

**Keywords:** antibiotics, amphenicols, detection, sensitivity, aquatic products, HPLC–MS/MS

## Abstract

Antibiotic drug residues are crucial to ensure food safety and minimize risk to human health. Herein, a sensitive high-performance liquid chromatography–tandem mass spectrometry (HPLC–MS/MS) method was developed and validated for the determination of antibiotic residues (mainly amphenicols) consisting of chloramphenicol (CAP), thiamphenicol (TAP), florfenicol (FF), and florfenicol amine (FFA) in aquatic products. Amphenicols were well separated on a Kinetex F5 (100 mm × 3.0 mm, 2.6 µm) chromatographic column with the mobile phases of 1 mM ammonium acetate aqueous solution and methanol solution and measured after positive and negative electrospray ionizations using four internal standards. To our knowledge, it was the first time to report the good performance of F5 column and four internal standards for the determination of amphenicols. The established method featured a good linear relationship between chromatographic peak area ratios and the concentrations of amphenicols (R^2^ > 0.992), a wide and low detection matrix-based range of 0.01–5 μg/L, a low detection limit of 0.01 μg/kg, etc. The spiked assays evidenced the accuracy and reliability of the developed method with the recoveries between 84.0 and 105%, the intraday relative standard deviations (RSDs) over the range of 0.769–13.7%, and the interday RSDs over the range of 0.582–13.3%. Finally, the proposed method was applied to investigate amphenicol residues in various aquatic products, including fish, shrimp, crab, shellfish, and other aquatic species.

## 1. Introduction

The increasing demand for aquatic products promotes the sustainable development of fishery. According to the China Fishery Yearbook, the total production of aquatic products reached approximately 655 million tons in 2020, a slight increase of 69 million tons compared to 2019. To prevent the various diseases, improve resistibility, and increase productivity, antimicrobials (mainly antibiotics) are routinely utilized in the process of aquaculture, and 73% of antimicrobials sold worldwide are used in animals raised for food [1]. As a type of antibiotics, amphenicols (i.e., chloramphenicol (CAP), thiamphenicol (TAP), florfenicol (FF), and florfenicol amine (FFA)) have been widely applied in livestock due to their low cost, high efficiency, good practicability, and strong capability of inhibiting the growth of Gram-positive and -negative bacteria, etc. [2,3,4]. However, CAP has been banned by many countries for its high toxicity and side effects to human hematopoietic system (e.g., aplastic anemia) [5,6]. As the derivatives of CAP, TAP and FF have less toxic effects and higher antibacterial activity than CAP, whereas which are deemed to the restricted veterinary drugs for their inhibition of red blood cells and embryonic toxicity [7,8]. FFA is mainly produced by FF through its metabolism in animal-derived food, and is the evidence of the usage of FF is also limited to veterinary drugs [9,10].

A variety of amphenicol detection approaches have been extensively developed by immunosorbent assay [11,12], fluorescence sensors [13,14,15], electrochemical sensors [16,17,18,19], gas chromatography–mass spectrometry (GC/MS) [20,21,22], and high-performance liquid chromatography–mass spectrometry (HPLC–MS/MS), etc. [23,24,25,26,27,28,29]. Among them, HPLC–MS/MS technology has drawn substantial attention owing to its rapid response, good sensitivity, high accuracy, strong separation ability, satisfactory compatibility of positive/negative ion modes, and widespread application in complex samples, such as swine, bovine, poultry eggs, and chicken muscle [30,,31,,32,]. However, the accumulation of antibiotics in aquatic products even a trace amount of amphenicol residues is likely a potential threat to public health and requires more attention during consumption and diet [33,34,35]. Therefore, it is important to develop a sensitive and accurate method to detect the trace levels of amphenicols to ensure the quality and safety of aquatic products and reduce the hazards caused by amphenicols.

**Figure 1:**
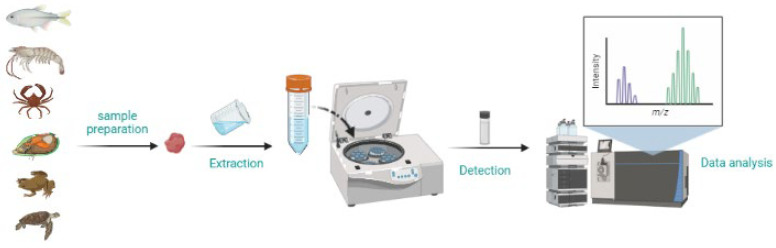
The schematic diagram of the proposed method.

Herein, we proposed a highly sensitive HPLC–MS/MS method for detecting the trace levels of amphenicol residues in various aquatic products (Figure 1). The liquid phase and mass spectrometric conditions were investigated comprehensively, including chromatographic column, mobile phase, and so on. The presented method was validated by linear detection range, spiked assays, etc. Finally, the proposed method was used for the determination of the trace amphenicols in various fish, shrimp, crab, and shellfish aquatic samples.

## 2. Results and Discussion

### 2.1. Mass Spectrometry Conditions

CAP, TAP, and FF displayed excellent response in negative ionization mode for the halogen atoms and hydroxyl groups, and FFA showed a favorable response in positive ionization mode, which was in agreement with the reported literature [36]. Additionally, the four internal standards were used to improve the accuracy of measurement in this work. Deprotonated molecular ions [M−H]^−^ were adopted as precursor ions for CAP (321.0 *m/z*), CAP-D5 (326.0 *m/z*), TAP (353.9 *m/z*), TAP-D3 (357.0 *m/z*), FF (356.0 *m/z*), and FF-D3 (359.0 *m/z*). Protonated molecular ion [M+H]^+^ was employed as precursor to analyze FFA (248.3 *m/z*) and FFA-D3 (251.3 *m/z*). Two different mass fragments were monitored for each external precursor, and the most abundant fragment ion was applied for quantification, and the other product ion was used for identification. Figure S1 shows the selective product ion chromatograms of the four amphenicols at the concentration of 25 ng/mL.

### 2.2. HPLC Conditions

#### 2.2.1. Chromatographic Columns

**Figure 2:**
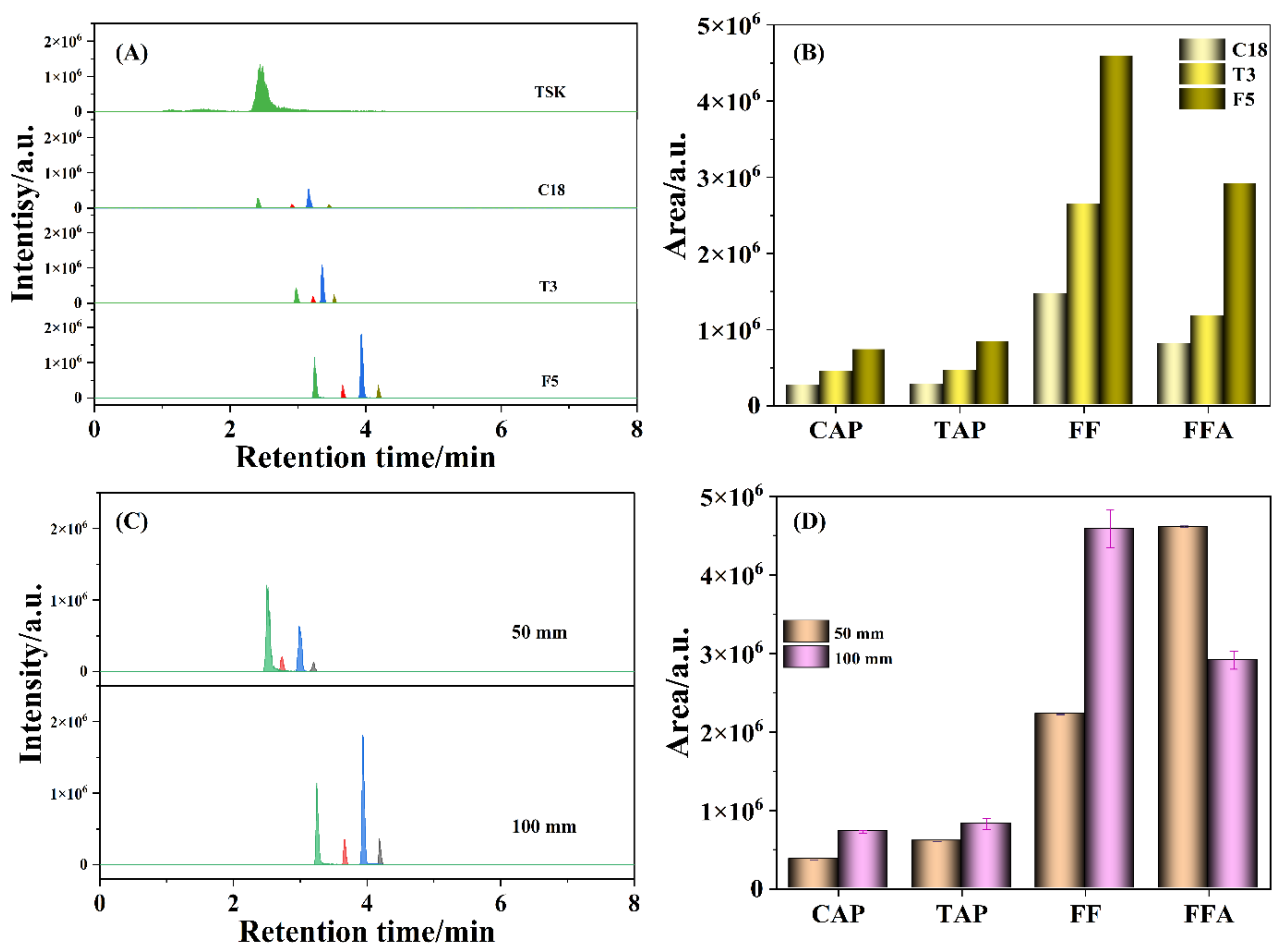
The chromatograms (**A**) and histograms (**B**) of peak areas for various chromatographic columns, the chromatograms (**C**) and histograms (**D**) of peak areas for different F5 chromatographic column length. Amphenicol standard solution: 50 ng/mL; mobile phases: 10 mM ammonium acetate and methanol; peaks from left to right: FFA, TAP, FF, and CAP.

The chromatographic columns have an important effect on the retention and separation of target compounds, which were investigated for better separation effect and higher sensitivity, including TSK-GEL Amide-80 (100 mm × 3.0 mm, 3 µm), ACQUITY HSS T3 (100 mm × 2.1 mm, 1.8 µm), CAPCELL PAK C18 (100 mm × 2.0 mm, 3 µm) and Kinetex F5 (100 mm × 3.0 mm, 2.6 µm). An amino chromatographic column is capable of separating compounds containing amino groups for the carbamoyl-bonded silica gel filler with suitable polarity, good retention, and high response [37]. As shown in Figure 2A, FFA fragment ion was well retained on the TSK-GEL Amide-80 column, but the other three amphenicols (i.e., CAP, TAP, and FF) were not retained, suggesting that the amide column is not suitable for separating the four amphenicols. A reversed-phase chromatographic column (e.g., C18) is specified for separating CAP, TAP, and FF in the national standard (GB 20756-2006). As a type of C18 column, a T3 column allows analytes to easily enter the pore structure of filler materials, enhancing the retention of polar and hydrophobic molecules. Compared to the TSK-GEL Amide-80 column, the four amphenicols were successfully separated on the C18 and T3 chromatographic columns. Nevertheless, the chromatographic peak of FFA was largely suppressed on the C18 and T3 columns, which was probably caused by the relatively poor retention of FFA on the C18 and T3 columns, leading to it eluting together with the dead volume [38]. Based on the pentafluorophenyl propyl filler, an F5 column could provide the unique polarity, hydrophobicity, aromaticity, selectivity of peak shape for halogen-containing molecules (e.g., F or Cl atoms), and satisfactory retention of the four amphenicols (the bottom line in Figure 2A). Moreover, it was observed that the chromatographic peak areas of the four amphenicols detected by the F5 column were higher than those by the T3 column and the stipulated C18 column (Figure 2B), which indicated that the ability to reserve amphenicols on the column was greatly enhanced, especially FF and FFA, thereby facilitating the improvement of detection sensitivity. To our knowledge, there are few reports with regard to the usage of an F5 chromatographic column for the separation and determination of antibiotic residues. Furthermore, the influence of the length of the F5 chromatographic column on the responses of CAP, TAP, FF, and FFA were evaluated simultaneously. As described in Figure 2C, the retention times of CAP, TAP, FF, and FFA detected with a 50 mm F5 column were 3.43, 2.95, 3.22, and 2.75 min, respectively, which were delayed to 4.19, 3.63, 3.94, and 3.26 min, respectively, with a 100 mm F5 column. Moreover, the chromatographic peak areas of most of the amphenicols tested with the 100 mm F5 column were higher than those with the 50 mm F5 column (Figure 2D). Therefore, the 100 mm F5 column was more suitable for the detection of amphenicols.

#### 2.2.2. Mobile Phase

**Figure 3:**
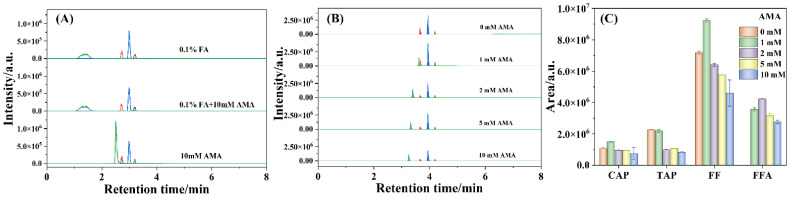
The chromatographic maps detected under different mobile phases (**A**) and varied concentrations of AMA (**B**) and the histograms of chromatographic peak areas (**C**) of the 50 ng/mL amphenicols. The peaks from left to right: FFA, TAP, FF, and CAP.

The composition of the mobile phase has a significant effect on the ionization efficiency of the analytes. Formic acid (FA) or acetic acid could provide a proton and always be adopted as the additive in the mobile phase to improve ionization efficiency [39]. Barreti et al. used ammonium acetate (AMA) as the additive in the mobile phase to improve FFA retention and reduce the interference caused by matrix components [38]. To study the effects of FA and AMA on the ionization efficiency of amphenicols, 0.1% FA aqueous solution, 0.1% FA aqueous solution containing 10 mM AMA (0.1% FA + 10 mM AMA), and 10 mM AMA aqueous solution were applied as the mobile phases in this work. As shown in Figure 3A, the shape of the FFA chromatographic peak with 10 mM AMA aqueous solution without FA as the mobile phase was much sharper than those obtained using 0.1% FA and 0.1% FA + 10 mM AMA aqueous solutions as the mobile phases, which suggested the relatively poor reservation of FFA on the F5 column in the presence of FA solution, implying the good performance of AMA on FFA reservation.

Meanwhile, the influences of AMA concentration on the responses of CAP, TAP, FF, and FFA were sequentially assessed, which was presented in Figure 3B,C. As observed in Figure 3B, FFA was hardly retained with the absence of AMA in the mobile phase (i.e., pure water) but was successfully retained as the concentration of AMA increased from 1, 2, 5, to 10 mM, further manifesting that AMA could promote the retention of FFA. Furthermore, the retention time of FFA shifted slowly from 3.62, 3.40, 3.33, to 3.25 min with the AMA concentration increasing from 1 to 10 mM, which illustrated that AMA was conducive to the separation of FFA. In addition, the retention times of CAP, TAP, and FF were not affected by the concentration of AMA even when the chromatographic peak areas were impacted. As shown in Figure 3C, the chromatographic peak areas of CAP, TAP, and FF fragment ions increased with the AMA concentration enhancing from 0 to 1 mM but decreased gradually with the AMA concentration further increasing from 1 to 10 mM, which was ascribed to the adduct formation with NH_4_^+^ at low AMA concentration in the electrospray [36]. Moreover, the peak area of FFA transition under 2 mM AMA aqueous solution was higher than those of other concentrations of AMA aqueous solutions. The results confirmed ionization enhancement for CAP, TAP, FF, and FFA at low concentration of AMA aqueous solution and ionization suppression at high concentration of AMA aqueous solution. Therefore, 1 or 2 mM AMA aqueous solution was appropriate for the detection of antibiotic residues.

**Figure 4:**
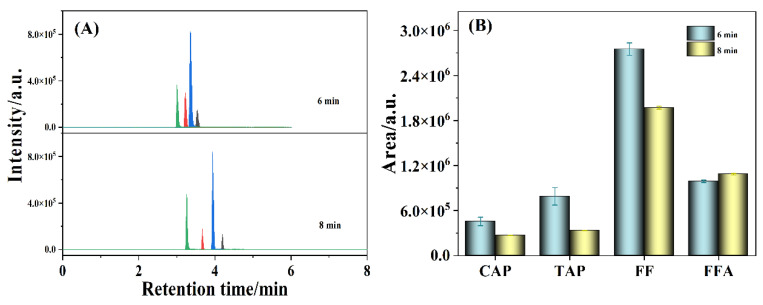
The chromatograms (**A**) and histograms (**B**) with error bar of 20 ng/mL CAP, TAP, FF, and FFA under different detection times. The peaks from left to right: FFA, TAP, FF, and CAP.

Additionally, to improve the rapidity of the detection method, the impact of detection time on the measurements was also studied, and the results are displayed in Figure 4. As depicted in Figure 4A, the retention times of CAP, TAP, FF, and FFA at a detection time of 8 min were 4.19, 3.67, 3.94, and 3.26 min, respectively, which were shortened to 3.54, 3.22, 3.36, and 3.00 min, respectively, at a detection time of 6 min. Moreover, the chromatographic regions of CAP, TAP, and FF at the detection time of 6 min were distinctly higher than those at 8 min, while the chromatographic region of FFA at the detection time of 6 min was fractionally smaller than that at 8 min (Figure 4B). Hence, in order to procure the high sensitivity and rapidity, a 6 min detection time was applied to measure the antibiotic residues.

Overall, the measurement of the amphenicol residues was performed on a 100 mm F5 column with the mobile phase of 1 mM AMA for 6 min. Figure S2 shows the chromatograms of 0.1 μg/L standard solution of amphenicols detected under the optimal conditions.

### 2.3. Method Validation

#### 2.3.1. Linearity, Sensitivity, and Matrix Effect of the Proposed Method

Under the optimized conditions, a facile, rapid, and sensitive method for amphenicol determination was established. Table 1 lists the parameters of the proposed method, such as the linear detection range, correlation coefficient (R^2^), limit of detection (LOD), limit of quantitation (LOQ), matrix effect (ME), etc.

It was observed that the ME values of CAP, TAP, FF, and FFA in *Carassius auratus* were more than 20%, 8.04%, less than −20%, and less than −20%, respectively, which indicated the ionic enhancement of CAP and the significant ionic suppression of FF and FFA in the *Carassius auratus* matrix (ME less than ±20% [40]). Additionally, the ME values of CAP, TAP, FF, and FFA in *Litopenaeus Vannamei* sample were 6.04%, −11.3%, less than −20%, and 11.9%, respectively, which also showed the ionic suppression of FF in the *Litopenaeus Vannamei* matrix. However, the ME values of CAP, TAP, FF, and FFA in *Eriocheir sinensis* and *Sinonovacula constricta* samples were all less than −20%, which revealed the serious ionic suppression of amphenicols in the *Eriocheir sinensis* and *Sinonovacula constricta* matrices. Therefore, to overcome the matrix effects and improve the accuracy of quantification for amphenicols, matrix-matched calibration curves were utilized during the measurement of amphenicol residues.

Obviously, the relationship between the area ratios and their corresponding concentrations of amphenicols in several matrix samples showed satisfactory linearity (R^2^ > 0.992) with the concentration varying from 0.01, 0.02, 0.05, 0.1, 0.2, 0.5, 1, 2, to 5 μg/L. LOD and LOQ were measured as 0.01 (signal-to-noise ratio, S/N > 3) and 0.02 (S/N > 10) μg/kg, respectively, which were much lower than those stipulated in the National Standard of China (GB 20756-2006: 0.1 μg/kg for CAP and 1.0 μg/kg for TAP, FF, and FFA), demonstrating the high sensitivity of the developed method for amphenicol detection. Table 2 summarizes the measurement of amphenicols in aquatic products reported in the literature using the HPLC–MS/MS method, which further demonstrated that the presented method exhibited relatively low and wide linear range and high sensitivity for the detection of the four amphenicols mainly due to the favorable performance of the F5 column and appropriate mobile phase.

#### 2.3.2. Accuracy, Repeatability, and Feasibility of the Proposed Method by Spiked Assays

Blank samples of *Carassius auratus*, *Litopenaeus vannamei*, *Eriocheir sinensis*, and *Sinonovacula constricta* were selected for the spiked assays to explore the accuracy, repeatability, and feasibility of the proposed method. As shown in Table 3, the average recoveries (R) of 0.20, 0.50, and 2.00 μg/kg of the four amphenicols were in the range of 84.0–97.8% in *Carassius auratus*, 89.0–105% in *Litopenaeus vannamei*, 89.2–101% in *Eriocheir sinensis*, and 89.7–99.8% in *Sinonovacula constricta*, which indicated the high accuracy of the developed method. Figure S3 shows the chromatograms of the spiked 0.5 μg/kg amphenicols in *Carassius auratus*. Furthermore, the intraprecisions and interprecisions of the amphenicols were in the range of 0.769–13.7% and 2.15–13.3% in *Carassius auratus*, 2.26–12.1% and 0.778–8.68% in *Litopenaeus vannamei*, 1.43–6.61% and 0.582–4.34% in *Eriocheir sinensis*, and 1.22–9.22% and 0.911–4.69% in *Sinonovacula constricta*. The results indicated that the established method featured good accuracy, favorable repeatability, and satisfactory feasibility and could be applied in the detection of amphenicol residues in real aquatic samples.

### 2.4. Detection in Real Aquatic Samples

A total of 720 aquatic samples were tested according to the optimized conditions, including fish, shrimp, crab, shellfish, and so on. To ensure the accuracy of measurement, quality control experiments were simultaneously conducted, and the recoveries ranged from 93.4 to 101%, which illustrated the reliability of the test. As listed in Table S1, amphenicol residues were found in 58 aquatic samples with detection rate of 8.06%. Specifically, the prohibited antibiotic drug CAP was not detected in any of the collected samples, while the restricted antibiotic drug TAP was found in three fish samples with levels of 0.834–1.81 μg/kg, which showed that the usage of CAP and TAP decreased under the strict policy. However, FF was detected in 27 aquatic samples containing fish, shrimp, crab, shellfish, and other aquatic products. The amounts of FF were over the range of 0.0615–107 μg/kg, with the maximum value of FF in *Pelodiscus sinensis*. Likewise, FFA was found in 33 aquatic samples (mainly fish and other aquatic products) with detection values in the range of 0.261–243 μg/kg, and the maximum value of FFA was measured in *Monopterus albus*. Currently, the National Standard of China (GB 31650-2019) stipulates that the maximum residue limits (MRLs) of TAP and FF (sum of FF and FFA) in the muscle and skin of fish should be 50 and 1000 µg/kg, respectively. The amount of amphenicols detected in the samples was much less than their MRLs, which implied the relative safety of the collected samples. Nevertheless, monitoring is still required to prevent the potential hazard from relatively high detection levels.

## 3. Materials and Methods

### 3.1. Materials and Instrument

The certified standard substances of chloramphenicol (CAP, 99%), thiamphenicol (TAP, 99%), florfenicol (FF, 99.8%), florfenicol amine (FFA, 99.8%), and chloramphenicol-d5 (CAP-D5, 100 ppm) were purchased from Dr. Ehrenstorfer GmbH (Augsburg, Germany). Thiamphenicol-d3 (TAP-D3, 97%), florfenicol-d3 (FF-D3, 98%), and florfenicol amine-d3 (FFA-D3, 98%) were supplied by Toronto Research Chemicals company (TRC, Toronto, Canada). Methanol (MeOH, HPLC grade), ethyl acetate (EA, HPLC grade), and n-hexane (95%, HPLC grade) were obtained from J. T. Baker Chemical Products Trading Co., Ltd. (Phillipsburg, NJ, USA). Formic acid (FA, 98%), ammonium acetate (AMA, HPLC grade, 99%), and ammonium hydroxide (25–28%) were provided by Shanghai Aladdin Biochemical Technology Co., Ltd. (Shanghai, China), Shanghai Honeywell Trading Co., Ltd. (Shanghai, China), and Sinopharm Chemical Reagent Co. Ltd. (Shanghai, China), respectively. Ultrapure water (18.2 MΩ·cm^−1^) was produced by Milli-Q EQ 7000 (Millipore, Billerica, MA, USA) and used throughout the experiments. A 5500 QTRAP triple quadrupole mass spectrometer (Applied Biosystems SCIEX, Framingham, MA, USA) equipped with a liquid chromatography (LC–MS/MS, Shimadu, Kyoto, Japan) was employed to measure the amphenicol residues.

### 3.2. Samples

The aquatic products samples were collected from Fujian, Guangdong, Shanghai, Zhejiang, Shandong, and Henan Provinces, including crucian carp (*Carassius auratus*), white shrimp (*Litopenaeus vannamei*), Chinese mitten crab (*Eriocheir sinensis*), *Sinonovacula constricta*, *Pampus cinereus*, turbot (*Scophthalmus maximus*), mandarin fish (*Siniperca chuatsi)*, *Silurus asotus*, scallop (*Placopecta magellanicus*), *Portunus trituberculatus*, white amur bream (*Parabramis pekinensis*), snakehead (*Ophiocephalus argus*), grass carp (*Ctenopharyngodon idellus*), bighead carp (*Aristichthys nobilis*), silver carp (*hypophthalmichehys molitrix*), yellow-headed catfish (*Pseudobagrus fulvidraco*), sea perch (*Lateolabrax japonicus*), common carp (*Cyprinus carpio*), loach (*Misgurnus anguillicaudatus*), large yellow croaker (*Larimichthys crocea*), freshwater eel (*Anguilla japonica*), swamp eel (*Monopterus albus*), red swamp crayfish (*Procambarus clarkii*), *Macrobrachium rosenbergii*, dark striped puffer fish (*Takifugu obscurus*), turtle (*Pelodiscus sinensis*), bullfrog (*Lithobates catesbeiana*), etc. Each sample was cleaned, and the edible tissues were dissected, cut into pieces, homogenized, and then stored at −20 °C for further treatment.

### 3.3. Sample Pretreatment

2.000 (± 0.002) g of the homogenized aquatic product samples were accurately weighed into 30 mL centrifuge tubes. Then, 40 μL of the 10 ng/mL mixed internal standard solution, 15 mL ethyl acetate solution, and 0.45 mL ammonium hydroxide were added in sequence. After shaking vigorously, the stock solutions were subjected to ultrasound for 10 min and subsequently centrifuged at 4000 r/min for 5 min. The supernatant was collected into a flask, and the lower layer sample was extracted repeatedly. The combined supernatant was concentrated by rotary evaporation at 40 °C. The dried flask was dissolved with 3.0 mL n-hexane and 2.0 mL water, which was then transferred to a centrifugal tube and centrifuged at 4000 r/min for 10 min. After removing the organic layer, the residual solution was filtered by 0.22 μm aqueous phase filtration membrane for the determination of amphenicol residues by HPLC–MS/MS.

### 3.4. LC–MS/MS Conditions

The amphenicol residues were separated by a Kinetex F5 chromatographic column (100 mm × 3.0 mm, 2.6 µm) at 40 °C with a flow rate of 0.35 mL/min and an injection volume of 10 μL. 1 mmol/L ammonium acetate aqueous solution and methanol were used as the mobile phase A and B, respectively. The gradient elution program is listed in Table 4.

The amphenicol residues were acquired in multiple response monitoring (MRM) positive and negative ionization modes in electrospray source ion (ESI) at a spray voltage of 4500 V. CAP, TAP, FF, FFA, and their corresponding deuterium internal standard substances were fully scanned by first-level mass spectrometry (Q1). The formed [M−H]^−^ precursor ions for CAP, TAP, and FF in ESI^−^ mode and [M+H]^+^ precursor ion for FFA in ESI^+^ mode were scanned by second-level mass spectrometry(Q3). The temperature of the ion transport tube was set to 650 °C. The mass spectrum parameters, such as curtain gas, ion source gas, and auxiliary gas, were optimized to appropriate values. The Q1 mass fragments, Q3 mass fragments, declustering potentials (DP), and collision energies (CE) of the amphenicol analytes are shown in Table 5.

### 3.5. Method Validation

The developed method was validated by linear range, calibration curves, correlation coefficient, LOD, LOQ, matrix effect, accuracy, etc. The calibration curves were plotted by the external standard to internal standard chromatographic peak area ratios and the corresponding concentrations of the standard solutions. The solvent-based calibration curve was prepared by water, while the matrix-based calibration curves were prepared by the blank matrix solution, which was acquired through pretreatment. LOD and LOQ were determined from the matrix-based calibration curve at S/N of 3 and 10 according to the reported literature, respectively [44,45]. ME was tested by the solvent-based and matrix-based calibration curves according to Equation (1) [46].

(1) $$ME\;\left(\%\right)=\left(1-\frac{S_{solvent}}{S_{matrix}}\right)\times 100\%$$

where S_solvent_ and S_matrix_ are the slopes of the solvent-based and matrix-based calibration curves, respectively. The value of ME implies an ionization enhancement (less than 0), an ionization suppression (more than 0), or no influence (equal to 0).

Spiked assays were utilized to investigate the accuracy, repeatability, and feasibility of the proposed method. The spiked levels were 0.2, 0.5, and 2 μg/kg, respectively. Each sample was conducted in three parallel tests to ensure the reliability of the results.

### 3.6. Statistical Analysis

All the figures were drawn by Origin 2021 software. Microsoft Excel 2019 was employed for statistical analysis, including calculation of the spiked recovery, relative standard deviations, uncertainty, etc.

## 4. Conclusions

A facile, rapid, and sensitive HPLC–MS/MS method was established for the determination of trace antibiotic residues (i.e., chloramphenicol, thiamphenicol, florfenicol, and florfenicol amine), and the level of antibiotic residues in various 720 aquatic products was studied. The liquid phase and mass spectrometric conditions were optimized, including chromatographic column, column length, mobile phase, and so on. The results showed that the F5 chromatographic column exhibited better performance than the TSK, T3, and C18 columns. Furthermore, 1 mM aqueous was the most appropriate mobile phase to promote the retention and separation of most components of antibiotic residues. Under optimal conditions, the proposed method displayed a good linear relationship in the matrix-based range of 0.01–5 μg/L and a low LOD of 0.01 μg/kg. The accuracy and reliability of the developed method were evidenced by the average recoveries ranging from 84.0 to 105%, intraday RSDs from 0.769 to 13.7%, and interday RSDs from 0.582 to 13.3%. Finally, the developed method was applied to detect amphenicol residues in real aquatic samples. The results showed that CAP was not detected in any of the collected samples, while the levels of TAP, FF, and FFA were over the ranges of 0.834–1.81, 0.0615–107, and 0.261–243 μg/kg in the acquired samples, respectively. The maximum values of TAP, FF, and FFA were tested in *Parabramis pekinensis*, *Pelodiscus sinensis*, and *Monopterus albus*, respectively. Although the levels of amphenicols were much less than their MRLs, monitoring is still needed to avoid the potential hazard of their bioaccumulation.

## Figures and Tables

**Table 1:** Linear range, LOD, LOQ, and matrix effect of CAP, TAP, FF, and FFA.

Matrix	Targets	Linear Range (μg/L)	R^2^	LOD (µg/kg)	LOQ (µg/kg)	ME (%)
H_2_O	CAP	0.01–5.00	0.99311	-	-	-
	TAP		0.99449			-
	FF		0.99579			-
	FFA		0.99613			-
*Carassius auratus*	CAP	0.01–5.00	0.99475	0.01	0.02	20
	TAP		0.99554			8.04
	FF		0.99592			<−20
	FFA		0.99642			<−20
*Litopenaeus Vannamei*	CAP	0.01–5.00	0.9959	0.01	0.02	6.04
	TAP		0.99353			−11.3
	FF		0.99362			<−20
	FFA		0.99675			17.9
*Eriocheir sinensis*	CAP	0.01–5.00	0.99568	0.01	0.02	<−20
	TAP		0.99592			<−20
	FF		0.99785			<−20
	FFA		0.9967			<−20
*Sinonovacula constricta*	CAP	0.01–5.00	0.99886	0.01	0.02	<−20
	TAP		0.99696			<−20
	FF		0.99442			<−20
	FFA		0.99275			<−20

**Table 2:** Comparison of amphenicol determination reported in the literature using the HPLC–MS/MS method.

Analytes	Columns	Mobile Phases	Linear Range	LOD (µg/kg)	Matrix	Ref.
CAP, FF	C18	2 mM ammonium acetate and acetonitrile, both with 0.1% formic acid	0.5–20 µg/kg	0.15	Fish	[41]
FF	C18	0.1% formic acid in water and acetonitrile	5–50 µg/kg	5.0	shrimp muscle	[42]
CAP, TAP, FF, FFA	C18	Water and acetonitrile, both with 2 mM of ammonium acetate	−	CAP:0.13, TAP:5.45, FF:2.86, FFA:248.20	Fish	[38]
CAP, TAP, FF, FFA	C18	Double-distilled water and 0.1% acetic acid in acetonitrile	–	CAP:0.01 (shrimp and flatfish); TAP:0.09 (shrimp), 0.05 (flatfish); FF: 0.01 (shrimp), 0.005 (flatfish); FFA: 1.3 (shrimp), 1 (flatfish);	shrimp and flatfish	[39]
CAP, TAP, FF	C18	Water and methanol	CAP: 0.3–50, TAP: 1.5–100, FF: 0.5–20	CAP: 0.02, TAP: 0.06, FF: 0.02	fish muscles	[43]
CAP, TAP, FF,	C18	Water and methanol, both with 0.1% formic acid	0.1–500 µg/L	CAP: 0.4, TAP: 1.0, FF: 0.2	feces (pig, chicken, and duck)	[25]
CAP, TAP, FF, FFA	F5	1 mM ammonium acetate and methanol	0.01–5.0 µg/L	0.01	Fish, shrimp, crab, and shellfish	This work

**Table 3:** The recoveries and RSDs of CAP, TAP, FF, and FFA by the spiked assays.

Matrix	Analytes	Spiked Levels (μg/kg)	Measured Levels (μg/kg)	Accuracy and Repeatability		
				R (%, n = 3)	Intra-RSD (%, n = 3)	Inter-RSD (%, n = 3)
*Carassius auratus*	CAP	0.20	0.193 ± 0.071	96.5	13.7	5.69
		0.50	0.480 ± 0.055	96.1	10.8	1.74
		2.00	1.88 ± 0.017	93.9	2.82	1.28
	TAP	0.20	0.180 ± 0.068	90.0	12.4	10.0
		0.50	0.489 ± 0.031	97.8	3.70	3.31
		2.00	1.87 ± 0.025	93.4	6.06	1.13
	FF	0.20	0.168 ± 0.078	84.0	3.31	13.3
		0.50	0.455 ± 0.049	91.1	3.75	3.70
		2.00	1.88 ± 0.035	93.9	5.53	3.23
	FFA	0.20	0.192 ± 0.048	96.0	7.01	2.88
		0.50	0.461 ± 0.033	92.3	4.38	2.15
		2.00	1.88 ± 0.025	94.0	0.769	3.29
*Litopenaeus vannamei*	CAP	0.20	0.185 ± 0.053	92.5	5.16	4.22
		0.50	0.454 ± 0.049	90.9	8.83	4.75
		2.00	1.92 ± 0.038	96.0	8.68	1.96
	TAP	0.20	0.178 ± 0.080	89.0	12.0	8.25
		0.50	0.476 ± 0.041	95.1	6.40	5.27
		2.00	2.02 ± 0.021	101	3.46	0.939
	FF	0.20	0.207 ± 0.021	104	2.43	1.37
		0.50	0.476 ± 0.030	95.3	5.90	0.778
		2.00	1.90 ± 0.018	94.9	2.26	1.41
	FFA	0.20	0.210 ± 0.068	105	12.1	8.68
		0.50	0.490 ± 0.022	98.0	3.82	1.55
		2.00	1.96 ± 0.027	98.2	2.35	1.24
*Eriocheir sinensis*	CAP	0.20	0.185 ± 0.046	92.3	4.54	4.34
		0.50	0.458 ± 0.034	91.5	6.50	1.53
		2.00	1.95 ± 0.017	97.7	2.51	1.02
	TAP	0.20	0.199 ± 0.030	99.7	2.76	2.94
		0.50	0.446 ± 0.041	89.2	6.61	3.23
		2.00	1.99 ± 0.024	99.4	4.46	0.582
	FF	0.20	0.192 ± 0.025	96.0	2.76	1.40
		0.50	0.475 ± 0.025	95.0	2.55	3.68
		2.00	2.00 ± 0.028	100	5.29	3.54
	FFA	0.20	0.201 ± 0.025	101	2.99	1.61
		0.50	0.487 ± 0.018	97.3	1.86	0.590
		2.00	1.93 ± 0.021	96.6	1.43	1.49
*Sinonovacula constricta*	CAP	0.20	0.182 ± 0.049	91.2	8.40	3.35
		0.50	0.493 ± 0.033	98.7	1.22	3.15
		2.00	2.00 ± 0.018	99.8	2.86	1.89
	TAP	0.20	0.190 ± 0.029	94.8	1.69	2.83
		0.50	0.484 ± 0.026	96.9	3.84	3.09
		2.00	1.97 ± 0.020	98.3	1.47	1.47
	FF	0.20	0.192 ± 0.026	96.2	3.65	1.20
		0.50	0.494 ± 0.016	98.9	1.96	0.911
		2.00	1.98 ± 0.018	99.2	2.03	1.13
	FFA	0.20	1.85 ± 0.061	92.5	9.22	4.69
		0.50	0.449 ± 0.042	89.7	2.87	3.20
		2.00	1.99 ± 0.016	99.5	2.19	1.15

**Table 4:** Gradient elution program of the target analysts.

Time/min	Mobile Phase A/%	Mobile Phase B/%
0	98	2
0.5	98	8
2.0	20	80
3.0	20	80
3.5	98	2
6.0	98	2

**Table 5:** Mass spectral parameters for amphenicols.

Compounds	Q1 Mass (*m/z*)	Q3 Mass (*m/z*)	DP (V)	CE (V)
CAP	321.0	152.1 *	−106	−21
		256.9	−138	−15
TAP	353.9	184.9 *	−80	−28
		289.9	−80	−18
FF	356.0	336.0 *	−80	25
		184.9	−80	−27
FFA	248.3	230.2 *	80	18
		130.2	80	33
CAP-D5	326.0	157.0	−80	−24
TAP-D3	357.0	188.1	−80	−30
FF-D3	359.0	188.1	−80	−27
FFA-D3	251.3	233.2	80	15

* Quantitative ion.

## Data Availability

Data are available on reasonable request from the corresponding author.

## Supplementary Materials

The following supporting information can be downloaded at: https://www.mdpi.com/article/10.3390/antibiotics11101427/s1, Figure S1. The selective product ions chromatograms of 25 ng/mL CAP, TAP, FF and FFA; Figure S2. Q3 ions fragments of the 0.1 μg/L amphenicols standard solution detected by the optimal conditions; Figure S3. Q3 ions fragments of the spiked 0.5 μg/kg amphenicols in *Carassius auratus*; Table S1. The determination results of amphenicols in 58 positive aquatic products.
