# Supplementary material for: Highly Sensitive Determination of Antibiotic Residues in Aquatic Products by High-Performance Liquid Chromatography–Tandem Mass Spectrometry
Source: Antibiotics (Basel). 2022 Oct 17;11(10):1427. doi: 10.3390/antibiotics11101427 (PMC9598229; doi:10.3390/antibiotics11101427)
Supplement: Supplementary file 1 [file antibiotics-11-01427-s001.zip › antibiotics-1891725-supplementary.pdf]

antibiotics

MDPI

Article

Supplementary Materials

Highly Sensitive Determination of Antibiotics Residues in Aquatic Products by High–Performance Liquid Chromatography–Tandem Mass Spectrometry

Hongli Ye [1,2], Siman Li [1], Yinfeng Xi [1,2], Yongfu Shi [1], Xiaorui Shang [1,3], and Dongmei Huang [1,*]

[1] Laboratory of Aquatic Product Quality, Safety and Processing, East China Sea Fisheries Research Institute, Chinese Academy of Fishery Sciences, Shanghai, 200090, China

[2] Key Laboratory of Control of Safety and Quality for Aquatic Product, Ministry of Agriculture and Rural Affairs, Beijing, 100141, China;

[3] College of Food Sciences and Technology, Shanghai Ocean University, Shanghai 201306, China;

* Correspondence: huangdm@ecsf.ac.cn (D. H.)

Citation: Ye, H.; Li, S.; Xi, Y.; Shi, Y.; Shang, X.; Huang, D. Highly Sen-sitive Determination of Antibiotics Residues in Aquatic Products by High–Performance Liquid Chroma-tography–Tandem Mass Spectrome-try . *Antibiotics* **2022**, 11, 1427. https://doi.org/10.3390/antibiotics11101427

Academic Editors: Yongning Wu and Carlos M. Franco

Received: 15 August 2022
Accepted: 14 October 2022
Published: 17 October 2022

Publisher's Note: MDPI stays neu-tral with regard to jurisdictional [illegible]ished maps and institu-tional affiliations.

Copyright: © 2022 by the authors. Submitted for possible open access publication under the terms and conditions of the Creative Commons Attribution (CC BY) license (https://creativecommons.org/licenses/by/4.0/).

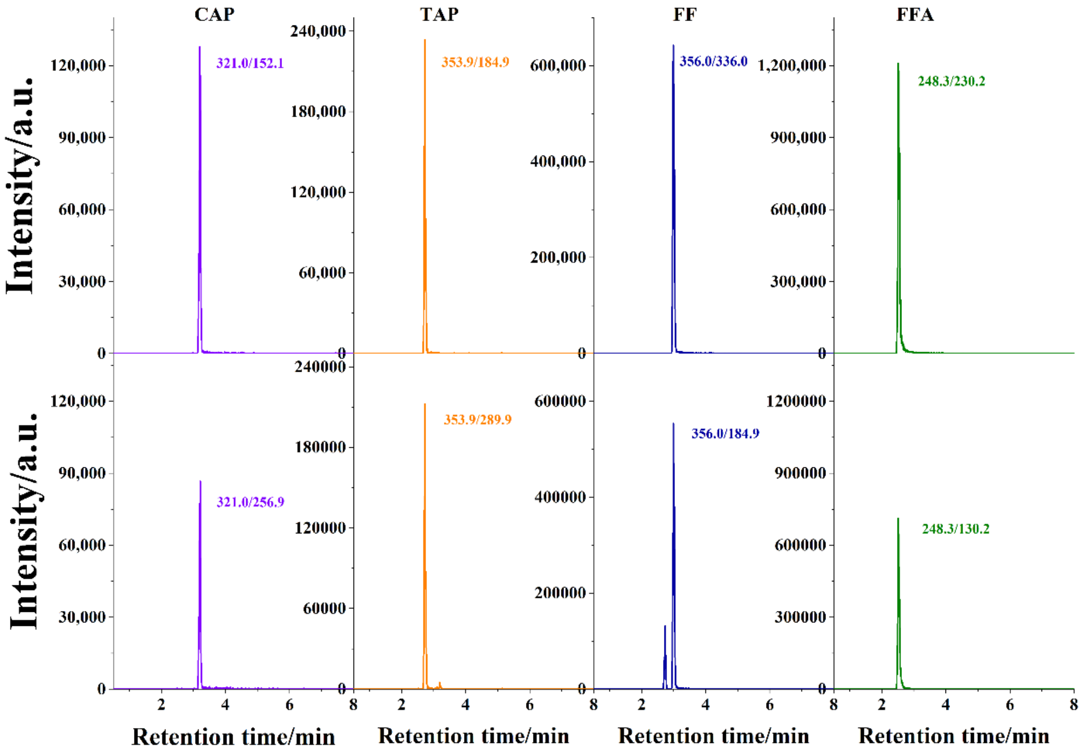

Figure S1. The selective product ions chromatograms of 25 ng/mL CAP, TAP, FF and FFA.

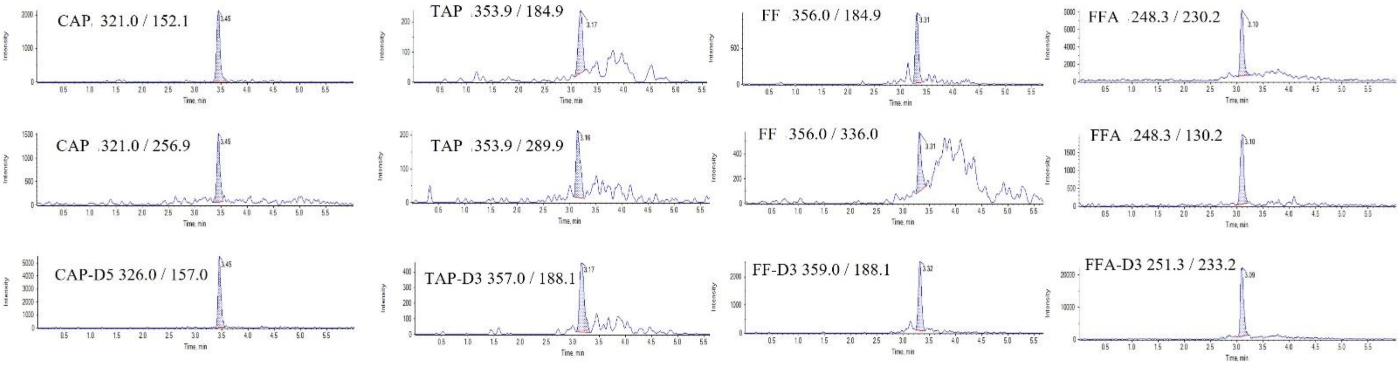

Figure S2. Q3 ions fragments of the 0.1 μg/L amphenicols standard solution detected by the optimal conditions.

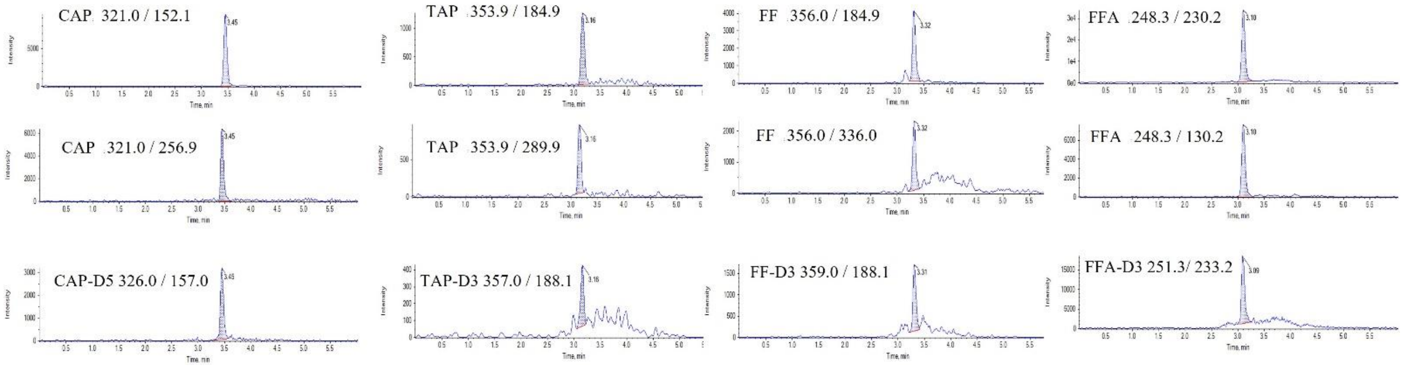

Figure S3. Q3 ions fragments of the spiked 0.5 μg/kg amphenicols in *Carassius auratus*.

Table S1. The determination results of amphenicols in 58 positive aquatic products.

Number	Species	Aquatic products	Measurement (μg/kg)			
			CAP	TAP	FF	FFA
1	Fish	*Silurus asotus*	N.D.	N.D.	0.171±0.021	N.D.
2		*Parabramis pekinensis*	N.D.	1.81±0.083	0.160±0.012	N.D.
3		*Ophiocephalus argus*	N.D.	N.D.	0.475±0.014	N.D.
4		*Hypophthalmichehys molitrix*	N.D.	0.977±0.057	N.D.	N.D.
5		*Lateolabrax japonicus*	N.D.	N.D.	0.113±0.006	0.261±0.015
6		*Larimichthys crocea*	N.D.	N.D.	0.0615±0.001	N.D.
7		*Anguilla japonica*	N.D.	0.834±0.015	N.D.	N.D.
8		*Monopterus albus*	N.D.	N.D.	4.56±0.021	N.D.
9		*Carassius auratus*	N.D.	N.D.	0.137±0.01	N.D.
10		*Lateolabrax japonicus*	N.D.	N.D.	2.94	47.8
11		*Ophiocephalus argus*	N.D.	N.D.	N.D.	43.1
12		*Ophiocephalus argus*	N.D.	N.D.	N.D.	9.81
13		*Aristichthys nobilis*	N.D.	N.D.	50.0	N.D.
14		*Ophiocephalus argus*	N.D.	N.D.	N.D.	27.0
15		*Aristichthys nobilis*	N.D.	N.D.	N.D.	14.1
16		*Scophthalmus maximus*	N.D.	N.D.	15.3	4.97
17		*Ophiocephalus argus*	N.D.	N.D.	N.D.	9.13
18		*Parabramis pekinensis*	N.D.	N.D.	6.07	N.D.
19		*Lateolabrax japonicus*	N.D.	N.D.	60.7	N.D.
20		*Ophiocephalus argus*	N.D.	N.D.	N.D.	97.2
21		*Lateolabrax japonicus*	N.D.	N.D.	N.D.	59.2
22		*Ophiocephalus argus*	N.D.	N.D.	N.D.	46.1
23		*Monopterus albus*	N.D.	N.D.	N.D.	243
24		*Monopterus albus*	N.D.	N.D.	N.D.	149
25		*Monopterus albus*	N.D.	N.D.	N.D.	78.3
26		*Monopterus albus*	N.D.	N.D.	N.D.	50.9
27		*Monopterus albus*	N.D.	N.D.	36.3	N.D.
28		*Ophiocephalus argus*	N.D.	N.D.	N.D.	106
29		*Lateolabrax japonicus*	N.D.	N.D.	N.D.	13.1
30		*Monopterus albus*	N.D.	N.D.	N.D.	89.0
31		*Scophthalmus maximus*	N.D.	N.D.	44.2	N.D.
32		*Parabramis pekinensis*	N.D.	N.D.	3.10	N.D.
33		*Cyprinus carpio*	N.D.	N.D.	31.2	N.D.
34		*Lateolabrax japonicus*	N.D.	N.D.	N.D.	14.2
35		*Cyprinus carpio*	N.D.	N.D.	N.D.	10.1
36		*Cyprinus carpio*	N.D.	N.D.	N.D.	18.8
37	shrimp	Procambarus clarkii	N.D.	N.D.	0.176±0.012	N.D.
38		*Macrobrachium rosenbergii*	N.D.	N.D.	1.96±0.029	N.D.
39		*Litopenaeus vannamei*	N.D.	N.D.	0.443±0.037	N.D.

40		*Litopenaeus vannamei*	N.D.	N.D.	*2.13*	N.D.
41	crab	*Eriocheir sinensis*	N.D.	N.D.	0.147±0.009	N.D.
42	shellfish	*Sinonovacula constricta*	N.D.	N.D.	0.618±0.004	N.D.
43		*Pelodiscus sinensis*	N.D.	N.D.	6.75	N.D.
44		*Pelodiscus sinensis*	N.D.	N.D.	107	N.D.
45		*Pelodiscus sinensis*	N.D.	N.D.	9.49	N.D.
46		*Pelodiscus sinensis*	N.D.	N.D.	11.8	N.D.
47		*Pelodiscus sinensis*	N.D.	N.D.	12.5	N.D.
48		*Pelodiscus sinensis*	N.D.	N.D.	N.D.	18.2
49		*Pelodiscus sinensis*	N.D.	N.D.	N.D.	118
50	other aquatic	*Pelodiscus sinensis*	N.D.	N.D.	N.D.	23.4
51	samples	*Pelodiscus sinensis*	N.D.	N.D.	N.D.	7.79
52		*Pelodiscus sinensis*	N.D.	N.D.	N.D.	23.5
53		*Pelodiscus sinensis*	N.D.	N.D.	N.D.	19.9
54		*Pelodiscus sinensis*	N.D.	N.D.	N.D.	16.1
55		*Pelodiscus sinensis*	N.D.	N.D.	N.D.	26.6
56		*Lithobates catesbeiana*	N.D.	N.D.	N.D.	18.0
57		*Pelodiscus sinensis*	N.D.	N.D.	N.D.	11.6
58		*Lithobates catesbeiana*	N.D.	N.D.	N.D.	10.6

* N.D.: the level less than LOQ
